# Polythiophene-Chitosan Magnetic Nanocomposite as a Highly Efficient Medium for Isolation of Fluoxetine from Aqueous and Biological Samples

**DOI:** 10.1155/2016/2921706

**Published:** 2016-09-08

**Authors:** Alireza Feizbakhsh, Amir Hossein Mohsen Sarrafi, Shokooh Ehteshami

**Affiliations:** Analytical Chemistry Laboratories, Department of Chemistry, Islamic Azad University, Central Tehran Branch, Tehran, Iran

## Abstract

Polythiophene/chitosan magnetic nanocomposite as an adsorbent of magnetic solid phase extraction was proposed for the isolation of fluoxetine in aqueous and biological samples prior to fluorescence detection at 246 nm. The synthesized nanoparticles, chitosan and polythiophene magnetic nanocomposite, were characterized by scanning electron microscopy, FT-IR, TGA, and EDAX. The separation of the target analyte from the aqueous solution containing the fluoxetine and polythiophene/chitosan magnetic nanocomposite was simply achieved by applying external magnetic field. The main factors affecting the extraction efficiency including desorption conditions, extraction time, ionic strength, and sample solution pH were optimized. The optimum extraction conditions were obtained as 10 min for extraction time, 25 mg for sorbent amount, 50 mL for initial sample volume, methanol as desorption solvent, 1.5 mL for desorption solvent volume, 3 min for desorption time, and being without salt addition. Under the optimum conditions, good linearity was obtained within the range of 15–1000 *μ*g L^−1^ for fluoxetine, with correlation coefficients 0.9994. Furthermore, the method was successfully applied to the determination of fluoxetine in urine and human blood plasma samples. Compared with other methods, the current method is characterized with highly easy, fast separation and low detection limits.

## 1. Introduction

**Figure 1:**
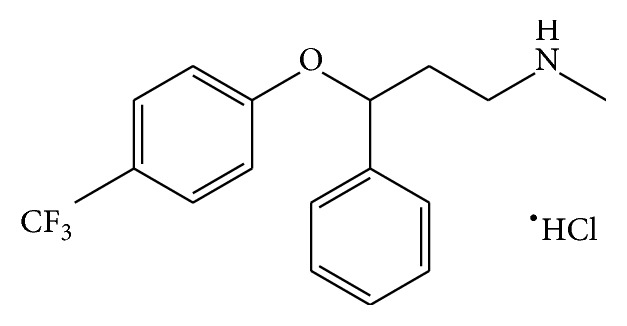
Structure of fluoxetine hydrochloric.

One of the most common serotonin specific reuptake inhibitors in neurons of presynaptic is N-methyl-3-phenyl-3-[4-(trifluoromethyl)phenoxy]propylamine hydrochloride or fluoxetine hydrochloride (Figure 1). It was developed since the late 1980s and is the most significant category of antidepressant drugs globally. Recently, fluoxetine was applied in the obsessive-compulsive treatment and eating disorders such as bulimia nervosa and anorexia. The acid dissociation constant (p*K*_a_) of fluoxetine is 8.7 as well as having log⁡*K*_ow_ of 1.0, 1.8, and 2.6 (pH values 5, 7, and 9) [1–3]. Elimination half-life of fluoxetine was ranged from one to several days, and less than 11% excreted unchanged fluoxetine while it is mainly excreted in urine [4]. A lot of fluoxetine rejection occurs in urine along with small amounts in the feces [5]. The dose of therapeutic could change from 20 to 60 mg per day related to the treatment and the levels of excreted urine are generally at mg L^−1^ [4]. In sight of the above considerations, the improvement of separation method for the fluoxetine determination from urine sample could be applied for toxicological and therapeutic purposes. Temporarily, due to high consumption of fluoxetine, a spreading threat of few residuals can occur into the environmental waters. The fluoxetine determination in biological samples is rather vital in human pharmacokinetic analysis, in drug's therapeutic checking, and in bioavailability/bioequivalence studies [1–4], while determination of fluoxetine in environmental water sample remains a drastic challenge in health issues. Diverse techniques have been applied for preconcentration and determination of trace amounts of fluoxetine in both aqueous and biological fluid phase samples. Different sample preparation methods such as liquid–liquid extraction [3, 6], solid phase extraction (SPE) [7], and stir bar sorptive extraction (SBSE) [8] along with chromatographic [3–9], spectrofluorimetric [3, 6, 10], and spectrophotometric instrument [6, 11] have been applied for determination of fluoxetine from biological and aqueous samples.

Although SPE technique was extensively used for various matrices, it has several drawbacks such as being time-consuming and tedious, needing too much organic solvents, and being relatively expensive. Lately, considerable attempts have been performed to develop new techniques to overcome these disadvantages. A new sample preparation derived of SPE is magnetic solid phase extraction that uses magnetic nanoparticles as adsorbents [12]. In this method, the sorbent does not demand to be filled into the SPE column; instead of it, the magnetic adsorbents are directly dispersed in the sample solution. Since the efficiency of extraction is enhanced because the contact interface between sorbents and analytes was increased, separation of phase can be easily performed from liquid phase to use an external magnetic field. Therefore, the magnetic sorbent can be quickly separated by a magnet and avoids the tedious centrifugation or filtration procedure [13–15].

These advantages of MSPE have caused that its applications in various fields, for instance, bioseparation, were increased [16–20]. However, the unmodified magnetic nanoparticles due to electrostatic and dipole-dipole interactions are easily accumulated and the surface area of particles was severely decreased. Therefore, the modification of their surface can not only enhance stability of magnetic particles in sample matrix but also offer a specific surface to interact with certain molecules such as organic and inorganic compounds [21, 22]. To date, several types of material such as organic polymers and inorganic materials have been used for alteration of reactivity of the magnetic nanoparticles. Among these modifiers, functional alkyl groups [23–25], organic polymers [26–29], graphene and graphene oxide [30–32], carbon nanotubes [33], and surfactants [34–36] are commonly investigated. Recently, conducting polymers (CPs) have been widely used as new coating materials [37, 38]. Polythiophene (PTh) compared to other CPs including polyaniline and polypyrrole has gained great attention because it is more stable than the others in high temperature and various solvents [39–41]. Yamini [40, 41] introduced a new MSPE sorbent based on PTh-coated Fe_3_O_4_. Fe_3_O_4_ nanoparticles do not have organic functional groups that can affect their adsorptive properties [42]. To overcome these limitations and increase the prominent merits of the magnetic materials, in this work, chitosan can be applied as a new modifier with the low aggregation properties during the growth of Fe_3_O_4_ nanoparticles. Therefore, chitosan can act not only as a “support” for the growth of Fe_3_O_4_ nanoparticles, but also as a “spacer” for the inhibition of nanoparticles aggregation.

For the first time, we developed polythiophene/chitosan magnetic nanocomposite as the MSPE technique for the extraction and analysis of fluoxetine in aqueous and biological sample. Firstly, magnetic nanoparticles and chitosan magnetic were synthesized through a facile coprecipitation, while different types of magnetic nanocomposites with different concentrations of thiophene (0.03–2 M) were coated on the surface of chitosan/Fe_3_O_4_ NPs by oxidized polymerization. The capability of these sorbents for extracting fluoxetine was examined. Extraction and desorption conditions were also optimized. To demonstrate the validation of the proposed method, the quantification limit, linearity, and precision were investigated.

## 2. Experimental

### 2.1. Reagents

Fluoxetine hydrochloride (99.37%) was obtained from Dr. Reddy Company (India). The fluoxetine standard solution (2000 mg L^−1^) was prepared with methanol (HPLC-grade) and stored at 2°C in refrigerator. Then, the working solution that contains concentrations was daily prepared via diluting the standard solution with double distilled water. Other chemicals such as sodium chloride, FeCl_3_·6H_2_O, FeCl_2_·4H_2_O, hydrochloric acid, sodium hydroxide, and methanol, ethanol, chloroform, dichloromethane, acetonitrile, and acetic acid were of analytical grades and were purchased from Merck (Darmstadt, Germany). The nitrogen gas (99.99%) was applied for providing the inert atmosphere necessary for synthesis of nanoparticles (NPs) and their coating process. Also, ammonium peroxydisulfate (APS) was obtained from Merck (Darmstadt, Germany) and was used without further treatment while thiophene (Mississauga, Canada) was distilled under reduced pressure.

### 2.2. Preparation of Magnetic Fe_3_O_4_ Nanoparticles

Fe_3_O_4_ NPs were prepared by coprecipitation of FeCl_3_ and FeCl_2_ [46]. Typically, 250 mL of sodium hydroxide solution with concentration of 1.5 M was prepared in ultrapure water under inert atmosphere at N_2_ gas. Then, an iron solution containing 5.2 g of FeCl_3_·6H_2_O and 2.0 g of FeCl_2_·4H_2_O as well as 0.85 mL of concentrated hydrochloric acid was freshly prepared in 25 mL of ultrapure water under inert atmosphere at N_2_ gas. Finally, the iron solution was added dropwise to the sodium hydroxide solution during 60 min with maximum vigorous stirring under a nitrogen atmosphere. The resulting black precipitates were separated from the reaction medium by a 1.4 T magnet and washed several times with degassed ultrapure.

### 2.3. Preparation of Chitosan-Coated Magnetic Nanoparticles

The chitosan magnetic nanoparticles (CS/MNPs) were prepared according to the following process. Firstly, 100 mL acetic acid aqueous solution (0.5% v/v) containing 5 g L^−1^ CS and FeCl_3_·6H_2_O (9.22 g) as well as FeCl_2_·4H_2_O (3.2 g) was freshly synthesized with maximum vigorous stirring at 45°C under the nitrogen atmosphere for 30 min. Then, the solution of sodium hydroxide (1.5 M) was added dropwise to the sodium hydroxide solution under maximum vigorous stirring in 30 min under an inert atmosphere of N_2_ gas. The hemimicelle of CS formed on the surface of magnetic nanoparticles and the solution color changed from orange to brown immediately. The resulting brown precipitates were separated from the previous medium using an external magnet. Finally, at room temperature, the suspension was washed sequentially with deionized water (3 × 100 mL) and methanol (3 × 100 mL) as well as ultrapure water (5 × 200 mL).

### 2.4. Preparation of Polythiophene/Chitosan Magnetic Nanocomposite

The preparation procedure of polythiophene/chitosan magnetic nanocomposite consisted of the following steps. First, chitosan/Fe_3_O_4_ submicrospheres containing functional groups (such as -OH and -NH_2_ groups) were prepared according to the above method. The hydrophobic and hydrophilic moieties of CS-MNPs could facilitate the dissolution of thiophene monomers. The next stage, the polythiophene/chitosan magnetic nanocomposite, was prepared by self-assembly polymerization method. Chitosan/magnetic nanoparticles (0.5 g) along with different amount of thiophene monomers were successively added into 25 mL of ultrapure water under maximum vigorous stirring in 30 min under an inert atmosphere of N_2_ gas at 60°C. Then suitable amount of APS, as initiator, was added to the solution and stirred for 1 h at this temperature. At this stage, the solution color was immediately changed from brown to black and polythiophene/chitosan magnetic nanocomposites were obtained. At room temperature, the resulting polythiophene/chitosan magnetic nanocomposite was collected by external magnet and washed several times by redispersion in methanol and double distilled water until the filtrate became colorless.

### 2.5. Extraction Procedure

**Figure 2:**
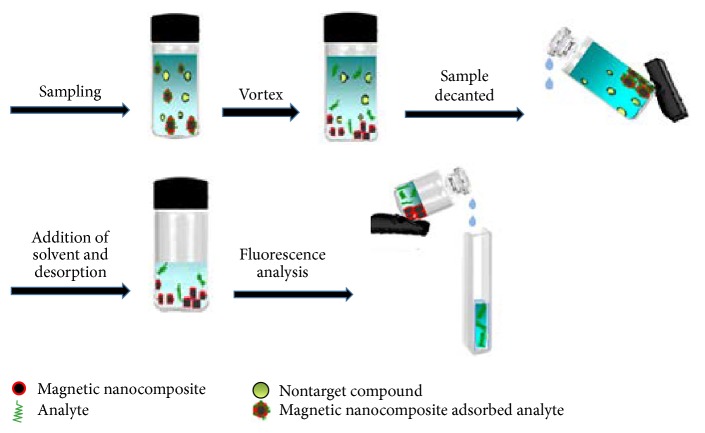
The whole extraction/desorption process setup.

For the magnetic solid phase extraction, 25 mg of polythiophene/chitosan magnetic nanocomposite was added into 50 mL double distillated water sample spiked with 1 mg L^−1^ of fluoxetine under the maximum stirring rate at room temperature for 10 min. Then, by applying external magnet on the outer wall of the vial, the polythiophene/chitosan magnetic nanocomposite could be easily collected and supernatant was removed. For desorption of fluoxetine from these magnetic sorbents, they were immersed in 1.5 mL of methanol for 3 min. Finally, the fluorescence spectrum of the desorbed solution was recorded by the spectrofluorimetric method. The extraction and desorption procedure was shown in Figure 2.

### 2.6. Real Sample Preparation

The human urine was obtained from the healthy volunteer and it is to be free of the selected drug. This urine sample was filtered using centrifugation at 10000 rpm for 10 min and the supernatant was fully collected. Then, the certain amount of fluoxetine standard solution was added into the urine supernatant and diluted (1 : 10 v/v) by double distillated water. Blank samples were prepared in the same way without the analyte-spiking step. Blood samples were obtained from a healthy individual and centrifuged at 10000 rpm. A plasma standard was prepared by adding the certain amount of fluoxetine standard solution to the diluted drug-free plasma solution to obtain the desired concentrations.

### 2.7. Instruments and Measurement

The EDAX analysis along with the scanning electronic microscopy (SEM) image was recorded by Cambridge Stereoscan 360 SEM Instrument (England) operating at 20 kV. Fourier transform infrared spectroscopy (FTIR) spectra were recorded using ABB Bomem MB100 (Quebec, Canada). Thermal stability of obtained sorbent was studied using thermogravimetric analysis (TGA): TG 209 F1 Iris-Thermogravimetric Analyzer from NETZSCH (Germany) at a heating rate of 20°C min^−1^ in the temperature range of 50–700°C under nitrogen atmosphere. The fluorescence spectra were registered by a Varian Cary Eclipse fluorescence spectrophotometer (Springvale, Victoria, Australia) equipped with 1 cm × 1 cm quartz cell and a xenon lamp. The fluorescence scan mode with slit widths of 5 nm was used for spectra recordings under excitation and emission wavelengths of 246 and 311 nm, respectively. The emission lines were recorded by A PMT detector at 600 V. The pH of sample was determined using a Metrohm Herisan pH meter (Switzerland). A 1.4 T magnet of NdFeB with model N48 (80 mm, 40 mm, and 30 mm) was purchased from Ningbo Strong Magnet Material Co., Ltd. (Ningbo, China).

## 3. Results and Discussion

In this work, a magnetic solid phase extraction based on a new magnetic nanocomposite sorbent for isolation of fluoxetine in a single step has been successfully developed. To achieve the maximum extraction efficiency of fluoxetine, several parameters such as volume and type of desorption solvent, sorbent amount, sample pH, adsorption, and desorption times and ionic strength of sample spiked with 1 mg L^−1^ of fluoxetine were optimized. Each experiment was performed in triplicate. To evaluate the influence of any factors, the peak intensity of extracted fluoxetine at 311 nm was considered.

### 3.1. Characterization of Polythiophene/Chitosan Nanocomposite

**Figure 3:**
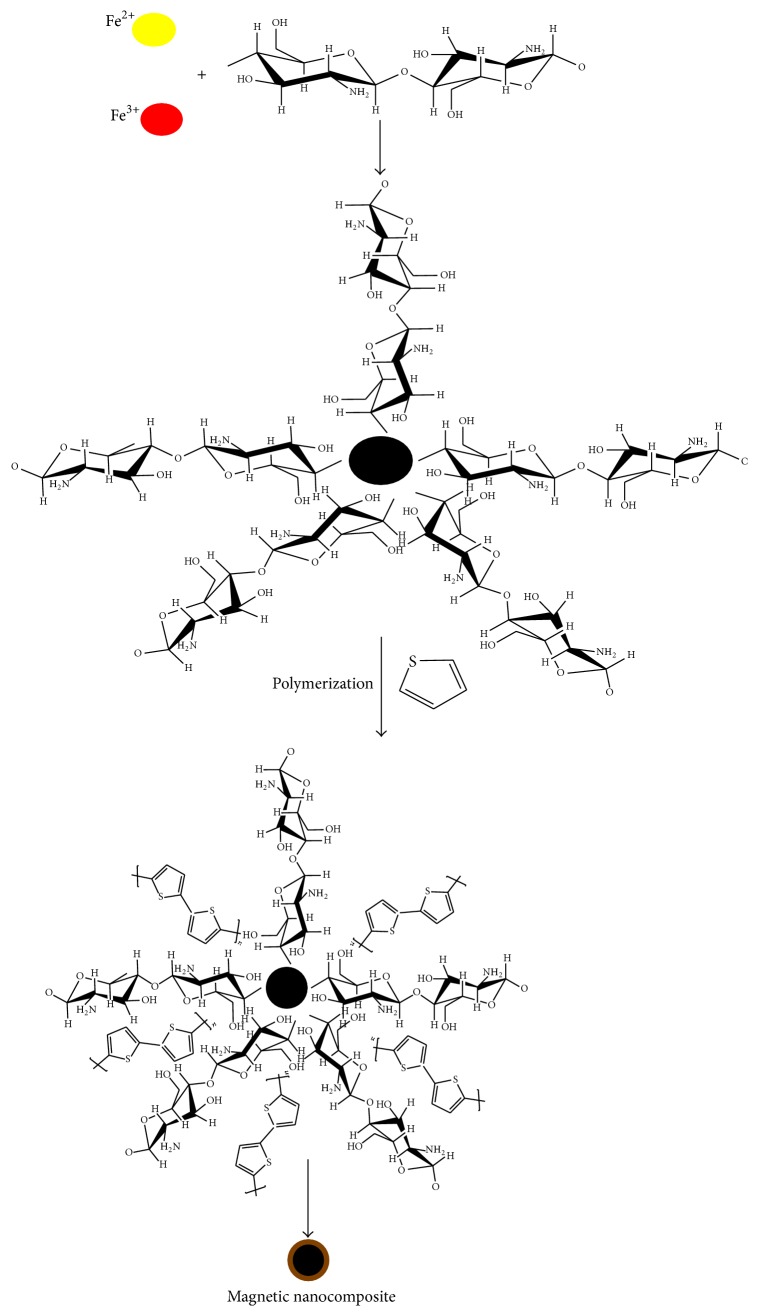
Schematic diagram for the preparation of CS-PTh magnetic nanocomposite.

The CS on the surface of magnetic nanoparticles played a significant role in the polythiophene-chitosan magnetic nanocomposites formation. Therefore, alkaline precipitation of aqueous solutions containing Fe (II) and Fe (III) at the present CS was performed. By adding sodium hydroxide, sample pH is increased to above 6.5 and the surface of Fe_3_O_4_ nanoparticles becomes negative. The charge of mineral oxides is negative when the pH value is above isoelectric point; while when the pH value is below this point then the surface of mineral oxides is positively charged. These negative charges on Fe_3_O_4_ nanoparticles, due to electrostatic attraction force with positive charge of -NH_2_ and -OH group in CS, could hold CS on the surface of them. Therefore, a complex of thiophene and CS-MNPs in aqueous solution was formed due to the acid-base and dipole-dipole interactions between -S group in thiophene monomers and the -OH and -NH_2_ group in CS. Finally, APS solution was added dropwise to the above solution and polymerization on the surface of the CS-MNPs takes place. Consequently, the “core” of CS-PTh magnetic nanocomposite is Fe_3_O_4_ nanoparticles while the “shell” of the prepared composite is CS-PTh (Figure 3).

**Figure 4:**
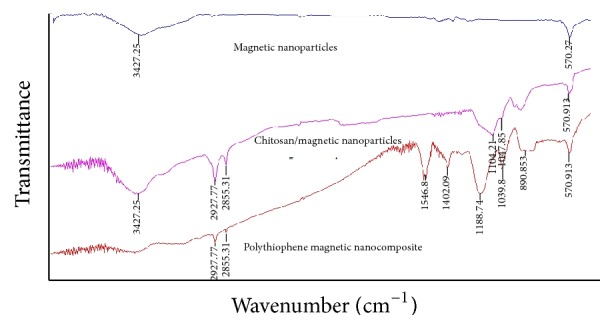
FTIR spectra of MNPs, CS-MNPs, and polythiophene-CS magnetic nanocomposite.

The FTIR spectra of MNPs, CS-MNP, and polythiophene-chitosan magnetic nanocomposite are shown in Figure 4. The stretching vibration peak of Fe-O at 570 cm^−1^ was observed in all the curves which confirmed the presence of Fe_3_O_4_ NPs in all synthesized materials. Moreover, in the spectrum of CS-MNPs was observed the characteristic absorption bands for CS at 2855 and 2927 cm^−1^ (C-H stretching vibrations), 3472 cm^−1^ (O-H and N-H stretching vibrations), and 1104 cm^−1^ (C-O-C stretching vibrations) along with one additional band at 570 cm^−1^ that corresponds to Fe-O. The FTIR spectrum of the polythiophene-chitosan magnetic nanocomposite was containing the Fe-O stretching vibration peak at 570 cm^−1^ related to MNP and the other characteristic absorption peaks of PTh such as band at 690 cm^−1^ for C-S stretching and the peaks at 2855 and 2927 cm^−1^ for the C-H stretching vibrations as well as peaks at 1546 and 1402 cm^−1^ that belong to C-C asymmetric and symmetric stretching vibrations of thiophene ring. Also, a peak observed in 1188 cm^−1^ is related to in-plane C-H aromatic bending vibrations of thiophene ring. All these proved that polythiophene-chitosan was coated on the surface of Fe_3_O_4_ NPs.

**Figure 5:**
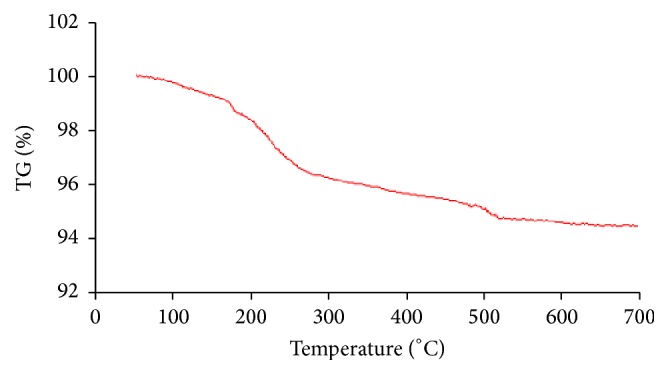
The data of TGA analyses for polythiophene-chitosan magnetic nanocomposite.

The TGA data of polythophene-chitosan magnetic nanocomposite in the range of 50–700°C was obtained under inert atmosphere of nitrogen. The TGA curve indicated a small initial weight loss under 160°C due to desorption of adsorbed water from the sample surface, followed by a continuous weight loss of the polythiophene-chitosan polymer coating layer that occurred above 160°C until 700°C due to decomposition of it (Figure 5). According to the weight loss, the amount of polythiophene-CS coating on Fe_3_O_4_ NPs was estimated to be approximately 5.8%.

**Figure 6:**
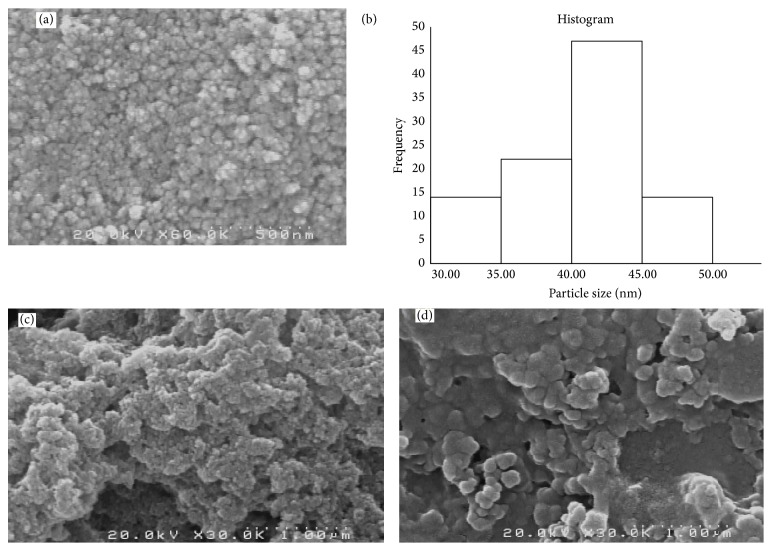
SEM images of magnetic nanoparticles (a), the histogram of nanoparticles based on the SEM image analysis of 100 particles (b), chitosan/Fe_3_O_4_ (c), and polythiophene-chitosan magnetic nanocomposite (d).

The morphology and structure of magnetic nanoparticles, chitosan magnetic and polythiophene/chitosan magnetic nanocomposite, were determined by SEM and FTIR. The SEM image of the dispersed iron oxide nanoparticles along with histogram plot shows that the synthesized MNPs have rather high surface area and the size of nanoparticles is less than 50 nm (Figures 6(a) and 6(b)). In this work, the synthesized Fe_3_O_4_/chitosan and polythiophene/chitosan magnetic nanocomposite (Figures 6(c) and 6(d)) have large specific surface area and are more suitable for absorption application.  Figure 6(c) shows that the final products exhibit slight aggregation as a result of surface modification by the attachment of PTh. This could be attributed to the fact that the reaction might occur on the particle surface and several PTh molecules were bound to chitosan on the magnetic particle.

**Figure 7:**
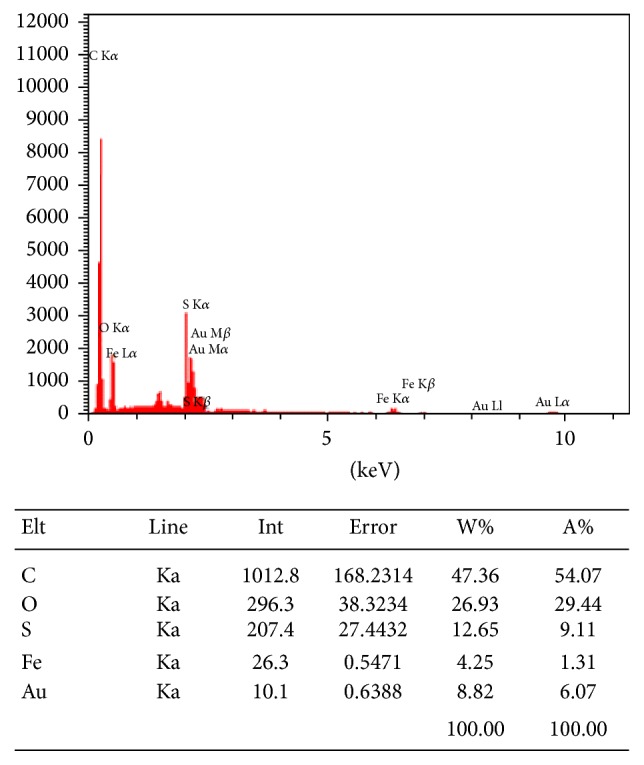
The EDAX spectrum of polythiophene-chitosan magnetic nanocomposite.

The EDXA spectrum of polythiophene-chitosan magnetic nanocomposite is also shown in Figure 7. The spectrum of polythiophene-chitosan magnetic nanocomposite indicates that the molar ratio of carbon, oxygen, sulfur, and iron are 47.36%, 26.92%, 12.65%, and 4.25%, respectively. It demonstrates that polythiophene was successfully coated on the chitosan/Fe_3_O_4_ nanoparticles.

### 3.2. Selection of Eluting Solvent

**Figure 8:**
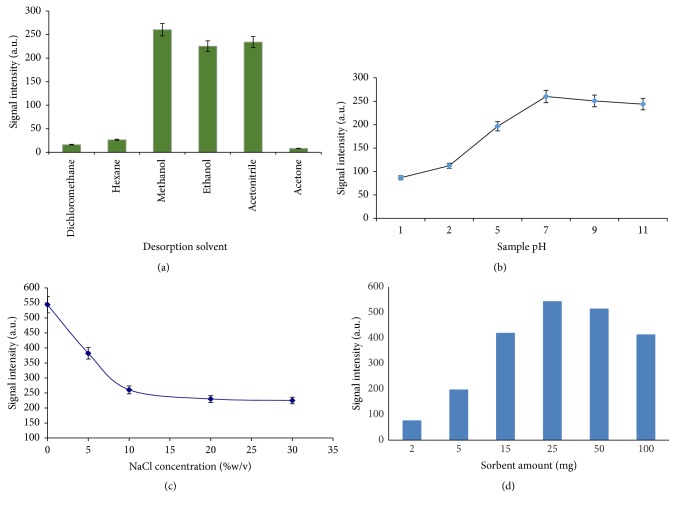
Effects of desorption solvent (a), ionic strength (b), sample pH (c), and sorbent amount (d) on the extraction efficiency.

**Figure 9:**
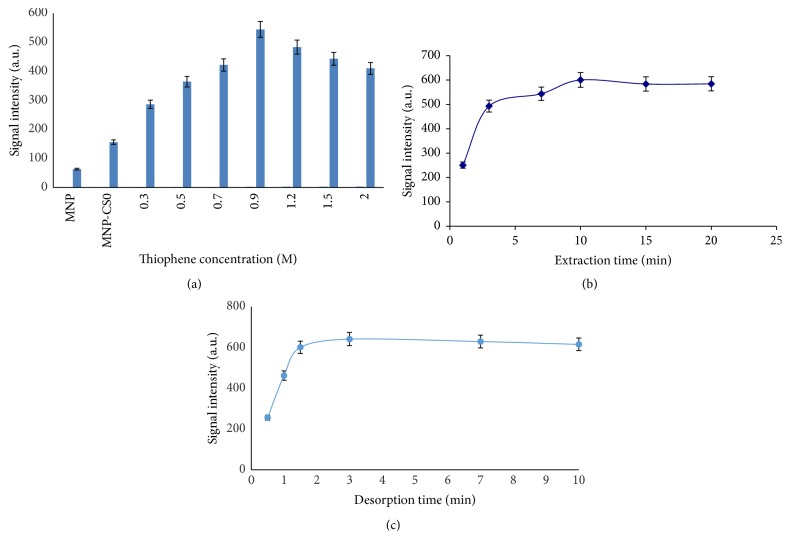
Effect of nanocomposite components ratio (a), extraction time (b), and desorption time (c) on the extraction efficiency.

The desorption solvent is an important parameter that has to optimized in the MSPE procedure. A minimum volume of suitable solvent can effectively desorb the analyte with less interfering impurities. In this experiment, six solvents, that is, ethanol, methanol, acetonitrile, dichloromethane, n-hexane, and acetone, were studied. The polar solvents such as acetonitrile, methanol, and ethanol provided good results (Figure 8(a)). However, there was maximum peak intensity when using methanol as eluting solvent. Therefore, methanol was selected. The solvent volume and desorption time were investigated. The volume of solvent increased from 0.5 to 2 mL (0.5 mL every time), and the desorption time from 0.5 to 10 min under maximum stirring rate was studied (Figure 9(c)). The time of desorption should be as short as possible while carryover effects must be considered comprehensively. The result indicated that desorption could occur completely within 3 min and 1.5 mL of methanol without carryover effect.

### 3.3. Sample pH

An influencing factor at the adsorption of polar compounds is solution pH that affects the charge of species and density on the sorbents surface. The effect of sample pH was considered from 1 to 11. The experimental results (Figure 8(b)) showed that the polythiophene/chitosan magnetic nanocomposite gave the best performance in neutral solution (pH = 7). This result can be easily described via the electrostatic forces between functional group of fluoxetine (-NH) and functional groups in polythiophene-chitosan magnetic nanocomposite (-NH_2_ and -OH at chitosan along with -S at thiophene) in the solutions having different pH values. Therefore, extraction efficiency of magnetic sorbent was probably reduced because the surface of the adsorbent (sulfur, oxygen, and nitrogen donor atoms) and fluoxetine (nitrogen donor atom) was protonated in strong acidic solutions. At this region (pH < 5) both of the analyte and sorbent have the same charge; an electrostatic repulsion force between them is expected to lower the extraction efficiency. As can be seen from the related figure further increase of pH above 7 resulted in slight decrease of extraction; thus, most likely pH 7 was selected because it provides the highest recoveries.

### 3.4. Ionic Strength

The ionic strength effect has been commonly used in various extraction techniques. Generally, addition of salt usually enhances the ionic strength of the aqueous solution and would affect the solubility of organic solutes. The increased ionic strength can reduce the concentration of water molecules available to dissolve solute molecules causing solutes to move faster into extracting medium. Therefore, the influence of ionic strength was considered in the range of 0–30% (w/v) of NaCl (Figure 8(c)). The effect of salting out was used in solid phase extraction and liquid–liquid extraction methods. However, the addition of salt had an adverse effect on the extraction efficiency of the magnetic solid phase extraction. This phenomena may occur due to the reduction of active sites on the magnetic sorbent or due to the increase in viscosity of the aqueous sample that is impeding the mass-transfer process. Consequently, the salt addition into water samples was abandoned.

### 3.5. Effect of the Amount of Magnetic Nanocomposite

The nanoadsorbents compared to microadsorbents have more satisfactory results because of their greater surface areas. To find the optimized amount of adsorbent, the amounts of PTh/CS magnetic nanocomposite were investigated from 2 to 100 mg. The results show that only 25 mg of magnetic sorbent was required to obtain maximum extraction of fluoxetine under the same conditions. When the amount was over 25 mg, all the PTh/CS magnetic nanocomposite may not effectively collect in the same time, which leads to a reduction in extraction efficiency. Furthermore, the minor amount of magnetic PTh/CS magnetic nanocomposite justifies the minimal volumes of elution solvent for efficient release of the analyte from the sorbent. Therefore, 25 mg of new sorbent was used in the next experiments, which was much less than the literature-reported amount of the commonly used microadsorbents.

### 3.6. The Effect of Components Ratio

The structure and morphology of adsorbent are crucial parameters in the extraction approach. In this work, the extraction efficiency of Fe_3_O_4_ nanoparticles coated with CS and different type of PTh/CS in various components ratios and undecorated magnetic nanoparticles were investigated by extracting fluoxetine, a model compound, from water samples. According to Figure 9(a) the extraction capabilities of the CS-MNPs are also higher than the undecorated iron oxide nanoparticles. Also, the effect of the thiophene concentration was considered in the range of 0.3–2 M, while CS-MNPs amount constantly remained at level of 0.5 g. The obtained results indicated that, by increasing the thiophene concentration until 0.9 M, the peak intensity was enhanced. Therefore, this trend conforms that PTh has an important effect in the extraction process and this concentration was chosen as the optimum value. By increasing the thiophene content in reaction solution, the magnetic properties of polythiophene/chitosan magnetic nanocomposite were decreasing and can lead to the loss of sorbent during their collection by the external magnetic field.

### 3.7. Effect of Extraction Time on the Adsorption Efficiency

The extraction time in the range of 1–20 min for the isolation of fluoxetine from water samples was studied (Figure 9(b)). The results of this experiment showed that the adsorption equilibrium time was achieved at about 10 min. The high surface area of MNPs along with uniform disperse of the sorbent throughout the sample could be the promising reasons for attaining such a fast extraction process. This is a superior benefit over the conventional SPE and other microextraction techniques, which generally need more than 30–60 min to touch the equilibrium. Consequently, an extraction time of 10 min was selected for the subsequent experiment.

### 3.8. Sorbent Reusability

Reusability is a considerable factor for estimating the efficiency of a magnetic sorbent. For determination of reusability, the extraction of the fluoxetine with the magnetic sorbent was performed for 25 times at the same condition. The obtained results indicated that the polythiophene-chitosan magnetic nanocomposite can be applied at least 25 times without a major loss of the extraction efficiency, while extraction efficiency after 30 times observed loss of 3%. Also, the reusability of the chitosan magnetic nanoparticles was considered and the result revealed that its extraction efficiency after 5 times extraction was reduced >14%. It shows that polythiophene induces a good stability to the CS-MNP. Also, stability of solvent is frequently associated with MSPE when it is coupled with derivatization techniques and/or liquid chromatography. Therefore, it is needed to immerse the polythiophene-chitosan magnetic nanocomposite directly into the appropriate solvent for 2 h. Solvent stability of the polythiophene-chitosan magnetic nanocomposite was considered to ensure that the synthesized coating has no bleeding during its contact with the organic solvent. The effect of several solvents with different polarities including water, dichloromethane, methanol, and acetonitrile was therefore considered. Two hours after sinking, extraction efficiency of polythiophene-chitosan magnetic nanocomposite at optimized condition was constant.

### 3.9. Analytical Performance

**Figure 10:**
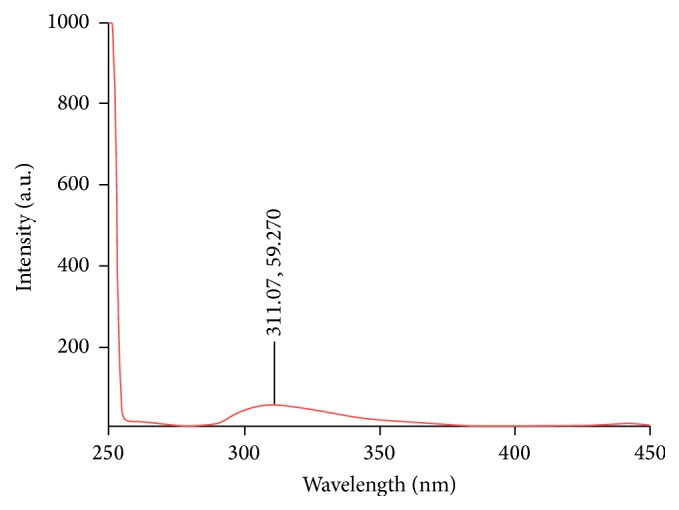
Fluorescence spectra of fluoxetine extracted from human urine sample with spiking fluoxetine at 75 *μ*g L^−1^.

After the optimization of important factors of magnetic solid phase extraction technique based on PTh/CS magnetic nanocomposite, some analytical features such as linear dynamic range, correlation of determination, limit of detection (LOD), limit of quantification (LOQ), and repeatability or relative standard deviation (RSD) were investigated. Analytical performance of the developed procedure is plotted in Table 1. Calibration curve via plotting peak intensity of each concentration versus associated concentrations of the fluoxetine in the range of 0.015–1 mg L^−1^ was obtained. The developed method exhibits a good linearity for fluoxetine throughout the concentration range with determination coefficient of *R* ^2^ = 0.9994. The LOD (S/N = 3) and LOQ (S/N = 10) values were 5 and 15 *μ*g L^−1^, respectively. The RSD value for five independent sample preparations was 2% at a concentration level of 75 *μ*g L^−1^. The reliability of current method was considered in various real samples such as tap water, human plasma, and human urine samples. The results in Table 1 confirm the validity of the proposed method. The fluorescence spectrum of extracted fluoxetine from urine sample spiked at 75 *μ*g L^−1^ is shown in Figure 10. The current work compared with several other methods on the determination of fluoxetine (Table 2) and the results indicated that it is either comparable or has rather pronounced advantages. Also, the results shown that the MSPE method based on new magnetic sorbent is a sensitive, easy, efficient, and reliable along with good analytical parameters in isolation of the fluoxetine from biological and aqueous samples. Furthermore, fast and cost-effective synthesis of the polythiophene/chitosan magnetic nanocomposite along with fewer amounts of sorbent and organic solvent as well as high stability in different solution is the vital advantage of the current method in comparison with other reports.

## 4. Conclusions

A novel type of modified magnetic nanoparticles coated with polythiophene was synthesized and employed as magnetic solid phase extraction adsorbent for isolation of fluoxetine in biological and aqueous water. Polythiophene/CS magnetic nanocomposite could be easily produced in large quantity using the oxidized polymerization method. The separation of fluoxetine-loaded magnetic adsorbent from the solution could be easily achieved via an external magnetic field. Moreover, the optimal method had attained acceptable analysis results of biological sample with a little amount of adsorbents within a short time. Coating of MNPs with polythiophene can not only increase adsorption ability of the target analyte, due to the existence of new interactions (hydrophobic and *π*-*π* interactions) between sorbent and target analyte, but also improve stability of the MNPs and their dispersibility in aqueous media.

## Figures and Tables

**Table 1:** Relative recoveries obtained for fluoxetine in different real samples.

Sample	Fluoxetine added (*μ*g L^−1^)	Fluoxetine found (*μ*g L^−1^)	Recovery (%)	RSD% (*n* = 3)
Tap water	0	0	—	—
	75	70	93	2

Urine	0	0	—	—
	75	42	56	4

Blood plasma	0	0	—	—
	75	58.5	78	3

**Table 2:** Comparing the current work with some other methods used for the determination of fluoxetine.

Method	Recovery (%)	LOD^a^ (*µ*g L^−1^)	LDR^b^ (*µ*g L^−1^)	RSD^c^ (%)	Analytical grade%	Triplicate	Ref.
Spectrofluorimetry	97	70	250–5000	2.2	99 purity	*n* = 5	[43]
LLE-CPE-FL	102	100	5000–50000	3.5	99 purity	*n* = 5	[43]
Spectrophotometry	19	—	1000–2000	1.0	99 purity	*n* = 5	[10]
SBSE-LC-MS	52–63	3	10–500	5.0	Analytical standards	*n* = 5	[8]
LLE-HPLC-FL	97–99	—	25–1000	1.0	99 purity	*n* = 5	[2]
SPE-CZE	89	10	100–200	3.0	—	*n* = 7	[5]
Magnetic-SPE- SF	80–104	20	50–1000	1.4	>99.3 purity	—	[35]
Magnetic-SPE- SF	76–99	1	50–5000	3	>99.3 purity	*n* = 3	[44]
Fluorimetric method	97–100	11	35–500	3	99.5 purity	*n* = 5	[45]
Developed method	56–93	5	15–1000	2			—

^a^Limit of detection.

^b^Linear dynamic range.

^c^Relative standard deviation.
