# Corrigendum to “Polythiophene-Chitosan Magnetic Nanocomposite as a Highly Efficient Medium for Isolation of Fluoxetine from Aqueous and Biological Samples”

**DOI:** 10.1155/2018/7265193

**Published:** 2018-04-01

**Authors:** Shokooh Ehteshami, Alireza Feizbakhsh, Amir Hossein Mohsen Sarrafi

**Affiliations:** Analytical Chemistry Laboratories, Department of Chemistry, Islamic Azad University, Central Tehran Branch, Tehran, Iran

In the article titled “Polythiophene-Chitosan Magnetic Nanocomposite as a Highly Efficient Medium for Isolation of Fluoxetine from Aqueous and Biological Samples” [1], the order of the authors was incorrect. The revised authors' list is shown above.
